# Impairments in proverb interpretation following focal frontal lobe lesions^☆^

**DOI:** 10.1016/j.neuropsychologia.2013.06.029

**Published:** 2013-09

**Authors:** Patrick Murphy, Tim Shallice, Gail Robinson, Sarah E. MacPherson, Martha Turner, Katherine Woollett, Marco Bozzali, Lisa Cipolotti

**Affiliations:** a National Hospital for Neurology and Neurosurgery, Box 37 Queen Square, London WC1N 3BG, UK; b Institute of Cognitive Neuroscience, University College, London, UK; c International School for Advanced Studies (SISSA), Trieste, Italy; d School of Psychology, University of Queensland, St. Lucia, Brisbane, Australia; e Department of Psychology, University of Edinburgh, Scotland, UK; f Dipartimento di Psicologia, University of Palermo, Italy; g Neuroimaging Laboratory, Santa Lucia Foundation, Rome 00179, Italy

**Keywords:** PFC, Proverbs, Executive function, Fluid intelligence

## Abstract

The proverb interpretation task (PIT) is often used in clinical settings to evaluate frontal “executive” dysfunction. However, only a relatively small number of studies have investigated the relationship between frontal lobe lesions and performance on the PIT. We compared 52 patients with unselected focal frontal lobe lesions with 52 closely matched healthy controls on a proverb interpretation task. Participants also completed a battery of neuropsychological tests, including a fluid intelligence task (Raven’s Advanced Progressive Matrices). Lesions were firstly analysed according to a standard left/right sub-division. Secondly, a finer-grained analysis compared the performance of patients with medial, left lateral and right lateral lesions with healthy controls. Thirdly, a contrast of specific frontal subgroups compared the performance of patients with medial lesions with patients with lateral frontal lesions. The results showed that patients with left frontal lesions were significantly impaired on the PIT, while in patients with right frontal lesions the impairments approached significance. Medial frontal patients were the only frontal subgroup impaired on the PIT, relative to healthy controls and lateral frontal patients. Interestingly, an error analysis indicated that a significantly higher number of concrete responses were found in the left lateral subgroup compared to healthy controls. We found no correlation between scores on the PIT and on the fluid intelligence task. Overall our results suggest that specific regions of the frontal lobes contribute to the performance on the PIT.

## Highlights

• Frontal lobe patients and controls were compared on a proverb interpretation task.
• Medial frontal lobe lesions are associated with deficits in proverb interpretation.
• Increased concrete responses in left lateral frontal patients.
• Impairments in proverb interpretation are not associated with fluid intelligence.

## 1 Introduction

### 1.1 Abstraction and the frontal lobes

The selective impairment of abstract thought processes as a result of neurological disease has long been known of; at least since such an impairment was held to reflect the loss of an *abstract attitude* (Goldstein, 1936, 1944). More specifically, it has been associated with lesions to the frontal cortex. Thus, Luria (1966, p. 285) concluded that the difficulties of frontal patients with abstraction emerged from their “lapse into irrelevant connections” when constructing abstract mental representations. As far as specific experimental results are concerned, Cicerone, Lazar, and Shapiro (1983) reported an abstract thought impairment in a group of frontal lobe patients who were shown to be unable to generate hypotheses regarding the underlying patterns in a visual learning task. Other studies showed that frontal lobe damage led to difficulties in abstracting rules in temporal and spatial patterns (Burgess & Shallice, 1996; Reverberi, Lavaroni, Gigli, Skrap, & Shallice, 2005; Villa, Gainotti, De Bonis, & Marra, 1990). More recently, using functional imaging, increased cerebral activity has been observed in prefrontal areas alongside increased “chunking” of specific visual sequences into abstract shapes during a spatial memory task (Bor, Duncan, Wiseman, & Owen, 2003).

Analogical reasoning is a further aspect of abstract reasoning that has also implicated the prefrontal cortex. In the non-verbal domain, fMRI studies have shown increased PFC activation when assessing the relationships between pairs of visual stimuli (Bunge, Helskog, & Wendelken, 2009; Krawczyk, Michelle McClelland, & Donovan, 2011), when deriving and applying rules from visual patterns (Hampshire, Thompson, Duncan, & Owen, 2011; Watson & Chatterjee, 2012) and when comparing the characteristics shared by pairs of people (Cho et al., 2010). In adolescents, a higher level of cortical maturity in prefrontal areas was found to reflect a better performance on a scene analogy task (Krawczyk et al., 2010). In the verbal domain, imaging studies have shown raised left prefrontal cortex activity when evaluating the relationships between semantic relationships (Bunge, Wendelken, Badre, & Wagner, 2005; Green, Fugelsang, Kraemer, Shamosh, & Dunbar, 2006; Green, Kraemer, Fugelsang, Gray, & Dunbar, 2010; Green, Kraemer, Fugelsang, Gray, & Dunbar, 2012). Event-related potentials have been used to find increased prefrontal activity for both a semantic analogical reasoning task (Maguire, McClelland, Donovan, Tillman, & Krawczyk, 2012) and a graphemic analogical reasoning task (Qiu, Li, Chen, & Zhang, 2008). However, despite this evidence, the specific localisation of processes supporting abstraction within the frontal lobes remains debated.

### 1.2 Proverbs and the frontal lobes

One of the most regularly used frontal lobe tasks tapping abstraction is proverb interpretation (see Gorham, 1956; Delis, Kaplan, & Kramer, 2001). As an aspect of figurative language, proverbs are familiar, fixed, sentential expressions that express well-known truths, social norms or moral concerns (Gibbs & Beitel, 1995). Common examples are *Rome wasn′t built in a day* and *All that glitters is not gold*. Proverb tasks assess the ability to interpret the proverbial statement in an abstract or metaphorical rather than a concrete sense, as the proverb’s meaning must be generalised to more scenarios than are reflected literally in the proverb itself (Delis et al., 2001, p. 205; Gibbs & Beitel, 1995). Impaired interpretations of proverbs have been regarded as an indicative of dysfunction in higher-level thinking processes linked with the frontal lobes. For example, Zeigarnik (1927), quoted by Luria, 1966 found evidence that patients with frontal lobe damage were unable to connect the literal and the metaphorical meanings of proverbs. Thus, one patient was noted as choosing the literal, rather than the correct abstract interpretation of the proverbs in a multiple-choice trial. Impaired proverb interpretation has been reported in other conditions affecting the frontal lobes such as Parkinson’s disease (Levin, Llabre, & Weiner, 1989). An increased tendency to interpret proverbs in a concrete sense in older people was found to be associated with decreased frontal executive skills (Albert, Wolfe, & Lafleche, 1990; Uekermann, Thoma, & Daum, 2008).

Despite their popularity as an assessment tool, relatively few studies have investigated the effects of focal cortical lesions on the ability to interpret proverbs. In an older study, where the basis for the classification of lesion location was not clear, Benton (1968) found that patients with bilateral frontal damage were significantly impaired in interpreting proverbs relative to those with unilateral damage. There was a trend towards a stronger performance in the left frontal group compared to the right frontal group with notable percentages of patients from each group (left frontal: 20%, right frontal: 25%, bilateral: 71%) performing in the impaired range.

In a more recent study, McDonald, Delis, Kramer, Tecoma, and Iragui (2008) compared patients with frontal lobe epilepsy (FLE), temporal lobe epilepsy (TLE) and healthy controls (HC’s) on a proverb interpretation task (PIT). To our knowledge, this is the only study where a systematic analysis of the errors made by frontal lobe patients on a PIT was undertaken. On an overall test score, FLE patients differed significantly from HC’s, but not from TLE patients in terms of proverb interpretation. In a multiple-choice trial, only FLE patients showed an increased tendency to choose concrete interpretations of the proverb. A further contrast based on the lateralisation of seizures showed that patients with left-sided FLE showed significantly poorer abstraction relative to the right-sided FLE patients.

Roca et al. (2010) reported data on a PIT from frontal lobe patients as part of a wider study of the nature of executive function deficits. The authors found that the frontal group as a whole was significantly impaired on proverb interpretation relative to HC’s. In a further group comparison, no difference was found between four different frontal subgroups: superior medial, inferior medial, left lateral and right lateral. Although a significant correlation was found between performance on the PIT and a test of fluid intelligence, patients and controls still differed on the PIT following adjustment for fluid intelligence. This indicates that proverb interpretation could not be entirely explained by a deficit in fluid intelligence. A subsequent lesion analysis examined the performance of six patients who performed the worst on five tests, including the PIT, where fluid intelligence did not account entirely for differences between patients and controls. This indicated that anterior (particularly right) frontal cortex was associated with the remaining deficits on these tests once the variance associated with fluid intelligence was accounted for. The authors suggested that lesions to this area may have damaged a “common processing theme” linking performance on these five tests.

### 1.3 Figurative language and the frontal lobes

The interpretation of proverbs, which undoubtedly requires the understanding of metaphor (Gibbs & Beitel, 1995), can be viewed as a part of the broader area of figurative language processes. Studies of brain damaged patients have provided evidence for frontal lobe involvement in such processes. Impaired idiom comprehension has been demonstrated following frontal lobe damage (Cacciari et al., 2006; Papagno, Curti, Rizzo, Crippa, & Colombo, 2006). This impairment has been shown to be associated with executive function deficits in Alzheimer’s disease (Papagno, Lucchelli, Muggia, & Rizzo, 2003). A reversed-concreteness effect was found for idiom comprehension in a patient with temporal lobe damage and a spared PFC (Papagno & Cacciari, 2010). In this study, interpretation of unambiguous and thus less figurative idioms was selectively impaired, with ambiguous idiom comprehension unimpaired. Impairments in metaphor processing have been associated with decreased activity in the left inferior frontal gyrus in patients with traumatic brain injury (Yang, Fuller, Khodaparast, & Krawczyk, 2010) and patients with schizophrenia (Kircher, Leube, Erb, Grodd, & Rapp, 2007), a condition linked with deficits in frontal executive functions (Minzenberg, Laird, Thelen, Carter, & Glahn, 2009).

In healthy subjects, neuroimaging studies have found associations between idiom comprehension and activity in left superior medial frontal gyrus and left inferior frontal gyrus (Romero Lauro, Tettamanti, Cappa, & Papagno, 2008), bilateral inferior frontal gyri (Zempleni, Haverkort, Renken, & Stowe, 2007), and left ventral dorsolateral PFC (Hillert & Buračas, 2009). One study utilising transcranial magnetic stimulation has indicated that left and right dorsolateral PFC is involved in idiom comprehension (Rizzo, Sandrini, & Papagno, 2007). Reading of metaphors has been associated with left inferior frontal gyrus activity (Rapp, Leube, Erb, Grodd, & Kircher, 2004; Stringaris, Medford, Giampietro, Brammer, & David, 2007).

There is ongoing debate regarding the role of left and right hemispheres in figurative language processing. Influential accounts have accorded a crucial role for right hemisphere regions through suppressing literal interpretations of figurative language or by processing less salient meanings (e.g. Bookheimer, 2002; Giora, Zaidel, Soroker, Batori, & Kasher, 2000; Stringaris et al., 2006; Tompkins & Lehman, 1998). However, these results are challenged by studies noting no effect of laterality on metaphor processing tasks (Rapp et al., 2004), greater deficits in pragmatics tasks in left-sided brain injury when compared to right-sided brain injury (Soroker et al., 2005) and an effect of congruity but not figurativeness on right-hemisphere processing of metaphors (Diaz & Hogstrom, 2011).

### 1.4 Aims

Findings from the literature support the view that the frontal lobes play an important role in proverb interpretation and in figurative language. However, questions remain regarding the localisation of processes within the frontal lobes that underpin the interpretation of proverbs. This study investigated this issue in a population of patients with unselected frontal lobe lesions performing a proverb interpretation task. Our primary goal was to examine if focal lesions to specific frontal lobe areas would have a differential effect on performance on the PIT and the pattern of errors made by patients. Following from Roca et al. (2010), we sought to establish if such deficits resulting from damage to specific frontal lobe areas could be explained by a deficit in fluid intelligence. A secondary goal was to establish if the performance of our patient group could shed further light on the role of right and left hemispheres in figurative language processes.

## 2 Materials and methods

### 2.1 Participants

Eighty-one patients with focal unilateral frontal lobe lesions were recruited from the National Hospital for Neurology and Neurosurgery (London, UK) and assessed in the Neuropsychology Department. The inclusion criteria for the study were: (i) age≤77 years, (ii) absence of psychiatric disorders, (iii) absence of other previous neurological disorders, (iv) absence of aphasia, (v) a score above the 5th percentile on a test of nominal functions, the Graded Naming Test (see below) and (vi) presence of a unilateral lesion confined to the frontal lobe documented by MRI or CT scan. Additionally (vii), patients were excluded if lesions were not localised to medial, orbital or left or right lateral areas of the frontal lobes as per the criteria outlined in Section 2.2 below. Fifty-two patients (*N*=52) met these criteria. Details of the aetiology of the lesions were available for *N*=48 patients, details of which are shown in Appendix A. The lesions were primarily the result of cerebrovascular accidents or tumour resections. Note that the data from these patients were gathered as part of a larger study of frontal lobe lesions. Patients from this study have been included in previous studies of frontal lobe dysfunction (MacPherson, Turner, Bozzali, Cipolotti, & Shallice, 2010; MacPherson et al., 2008; Robinson, Shallice, Bozzali, & Cipolotti, 2010, 2012; Turner, Cipolotti, Yousry, & Shallice, 2007).

Each patient was matched with a healthy control (HC) for age, gender and full-scale IQ as estimated by the National Adult Reading Test (NART). Fifty-two HC’s were included in the study from a group of 107 initially assessed. Both left and right frontal groups and left lateral, right lateral and medial frontal subgroups were compared with HC’s in terms of age, gender, NART full-scale IQ and performance on the Graded Naming Test. No significant differences were found (see Tables 1 and 2 for a summary of group and subgroup demographic details). The latter result indicates that there was no difference between the groups in terms of their pre-morbid intellectual functioning.

All participants gave informed consent according to the Declaration of Helsinki 1991. Our study was approved by the National Hospital for Neurology and Neurosurgery and the Institute of Neurology Joint Research Ethics Committee (UK). Other aspects of the performance of the participants in this study have been reported previously (MacPherson et al., 2008, 2010; Robinson et al., 2010, 2012; Turner et al., 2007).

### 2.2 MRI and lesion analysis

**Fig. 1:**
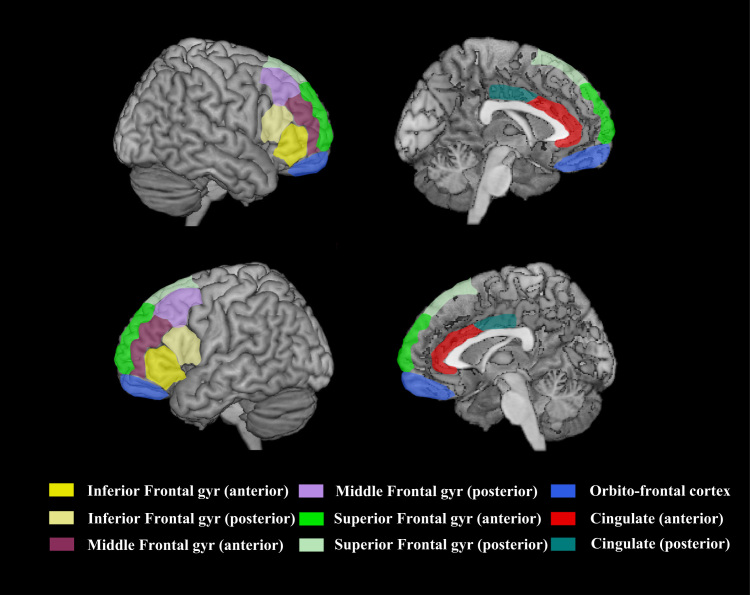
Frontal lobe areas used to partition patients into subgroups of interest.

**Fig. 2:**
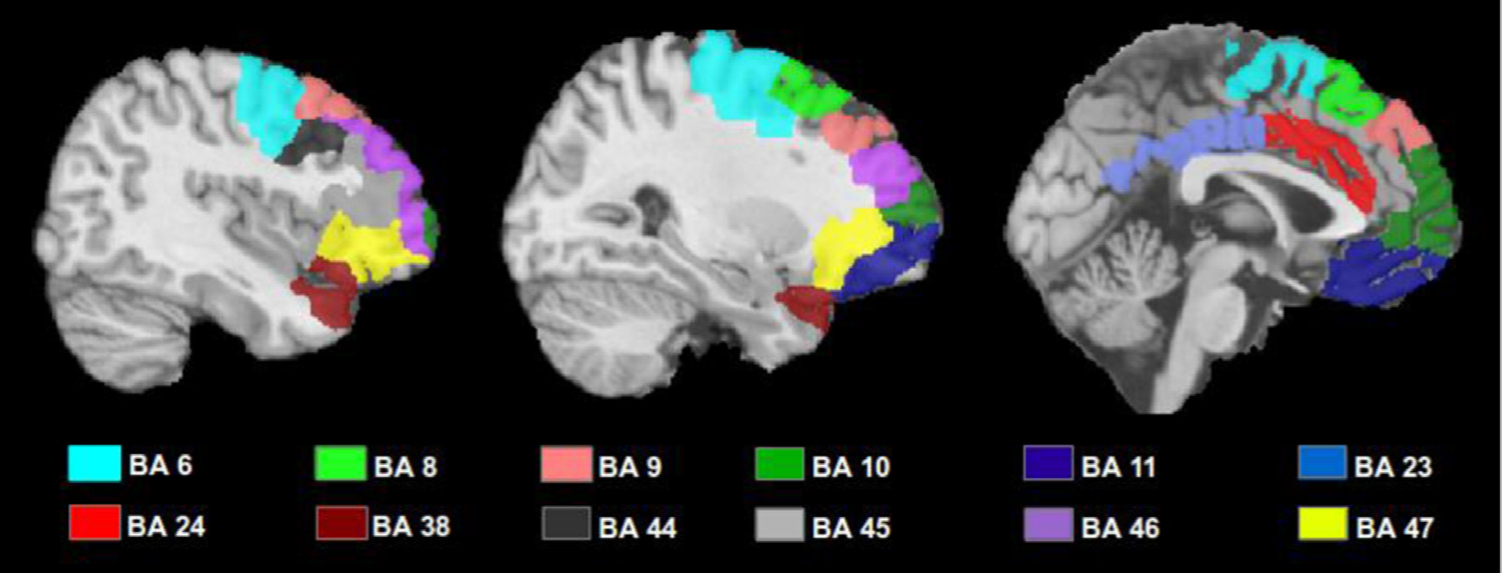
Depiction of Brodmann areas contained within subgroups of interest.

A neurologist who was blind to the history of each patient reviewed the hard copies of MRI scans (or CT scan where MRI was unavailable). Brain MRI was obtained on systems operated on a 1.5 T machine and included the acquisition of an axial dual-echo and an axial and coronal T1-weighted scan. CT scans were all obtained using a spiral CT system. Both MRI and CT data were used, as the main goal of the current study was to enable the recruitment of a large number of patients. The exclusion criteria and lesion assessment guidelines were based on detailed anatomical localisation using standard atlases (Duvernoy, 1991). The lesion localisation method is described in detail in Robinson et al. (2012). Briefly, each frontal patient was coded for the presence of lesion in each hemisphere in the anterior and posterior portions of nine left and right frontal regions (18 areas in total) (see Figs. 1 and 2). An area was only coded as damaged if at least 25% of the area was affected.

Two types of lesion analyses were carried out. The first analysis aimed at assessing the lateralisation effect by collapsing the nine left and right brain regions and dividing the patients into two groups, i.e., left and right frontal, according to which hemisphere was damaged.

In the second analysis, a more refined anatomical sub-division was employed, similar to that used in previous studies (e.g. MacPherson et al., 2010; Robinson et al., 2010, 2012; Turner et al., 2007). The prefrontal regions were collapsed in order to define three main subgroups of patients: medial, left lateral and right lateral.

**Fig. 3:**
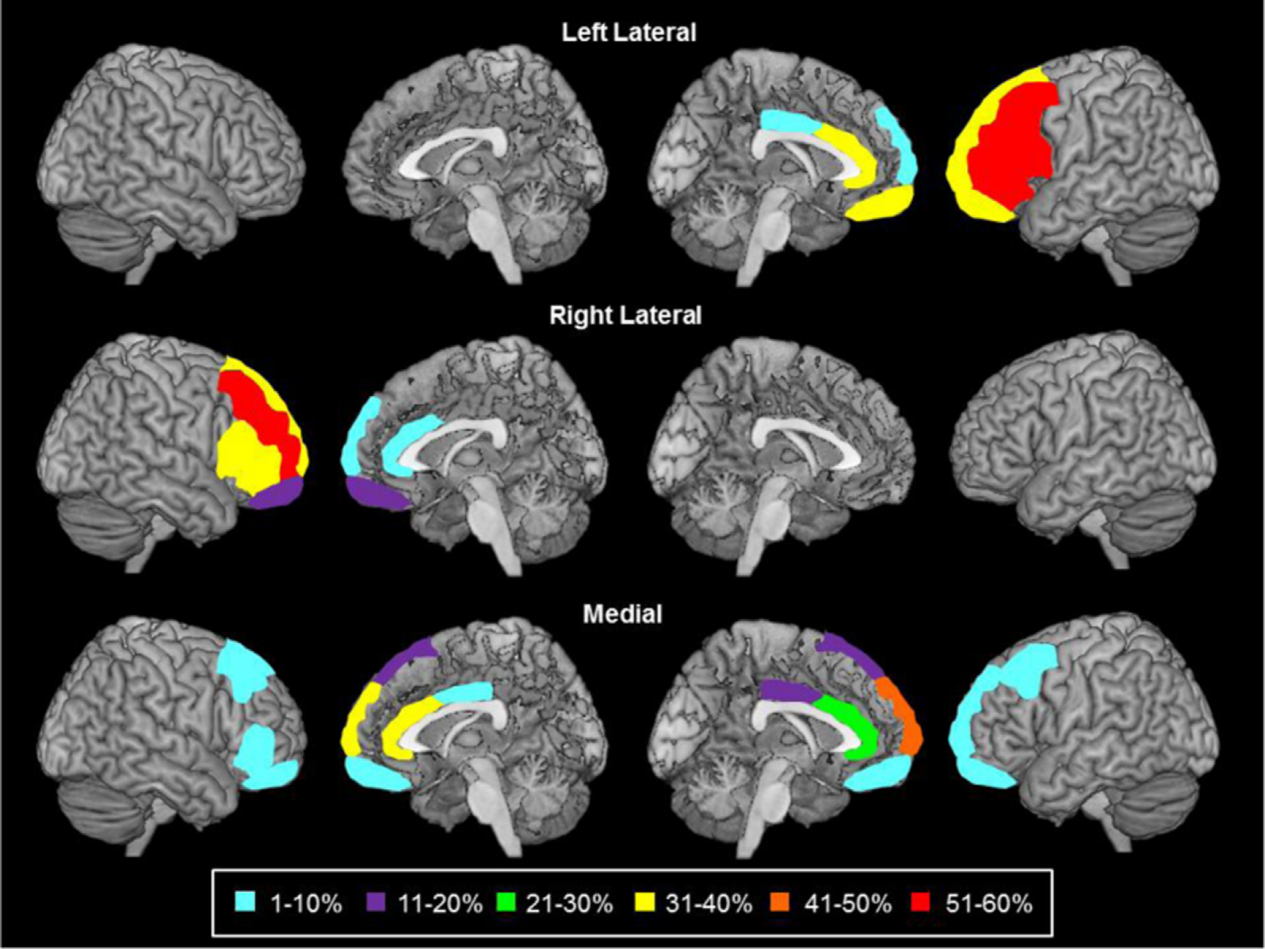
Damage to frontal lobe regions for each frontal patient subgroup. Shading describes percentage of patients from each group with damage to specified area.

For these subgroups, primary damage to the respective cortical areas was defined as either (a) damage that was restricted to the cortical regions that defined the subgroup or (b) damage that affected at least three cortical regions that defined the subgroup and no more than one other region that defined an adjacent subgroup. The medial subgroup (*N*=17) consisted of patients with unilateral primary damage to the left (Fig. 1, panel B) or right (panel D) cingulate gyrus (anterior/posterior), and/or the left (panel B) or right (panel D) medial superior frontal gyrus (anterior/posterior). These areas consist of or overlap with Brodmann areas 6, 8, 9, 10, 23, 24, 32 and 33. The lateral subgroups consisted of patients with primary damage to the left (*N*=16; panel C) or right (*N*=13; panel A) lateral part of the superior frontal gyrus (anterior/posterior), and/or the left (panel C) or right (panel A) middle frontal gyrus (anterior/posterior) and/or the left (panel C) or right (panel A) inferior frontal gyrus (anterior/posterior). These areas consist of or overlap with Brodmann areas 6, 8, 9, 38, 44, 45, 46 and 47. See Fig. 3 for percentage of patients from each group with damage to specified areas and Fig. 2 for depiction of Brodmann areas contained within subgroups of interest. A further group of patients with primary damage to the left (panels B and C) or right (panels A and D) orbital cortex (blue areas, Brodmann areas 10 and 11) were excluded from the analysis due to the small number of patients in the group (*N*=6) and the lack of error data available (half of the sample). However, a qualitative description of the orbital patients will be provided.

We found no difference between left and right hemisphere groups and between the medial, left lateral and right lateral subgroups in terms of the number of frontal regions damaged (see Tables 1 and 2). The lesion analysis also revealed that the lesions of 10 of our patients resulted in basal ganglia damage. The likelihood of basal ganglia damage did not differ across the frontal subgroups, *χ*^2^(3, *N*=46)=2.500, *p*=0.287.

### 2.3 Neuropsychological investigation—baseline cognitive tests

The battery comprised well-known clinical tests with published standardised normative data collected from large control samples. The National Adult Reading Test (NART) was administered to estimate pre-morbid optimal levels of functioning (Nelson & Willison, 1991). Raven’s Advanced Progressive Matrices (RAPM, Raven, 1976), an untimed, relatively culture-fair, non-verbal test, was used to assess abstract reasoning. The Graded Naming Test (GNT, McKenna & Warrington, 1980) was used to assess nominal functions. Executive functions were assessed using two tests known to be sensitive to frontal lobe dysfunction, namely the Stroop test (Trenerry, Crosson, DeBoe, & Leber, 1989) and the Trail Making Test (Reitan, 1958). For the Stroop test we recorded the number of words or the number of colours named in 2 min. For the Trail Making Test we report the results of Part B, known to be sensitive to executive dysfunction (Stuss et al., 2001).

### 2.4 Proverbs test (PIT)

The PIT was adapted from the the D-KEFS Proverb Test (Delis et al., 2001). The PIT contains eight metaphoric proverbs (see Supplementary material, Appendix A), used in the English (UK) language. Each proverb is read aloud to the subject, who provides an explanation of the meaning of the proverb. The participants’ responses are recorded by the clinician. This task assesses the ability to interpret a statement more in an abstract rather than a concrete sense, as the proverb’s meaning must be generalised to more scenarios than are reflected literally in the proverb itself (Delis et al., 2001, p. 205). For example, for the proverb “Rome wasn′t built in a day”, a generalised understanding is that any great achievement (“Rome”) takes patience and time to complete (“wasn′t built in a day”). A concrete or literal (i.e. less generalised) understanding of the proverb may refer to the *length of time* it takes to complete buildings or infrastructure or the *time it took to establish* the Roman Empire.

The first analysis assessed the *accuracy of interpretation* of the proverb (or “free enquiry”, see Delis et al., 2001). Each oral response to an item on the PIT was scored out of 2. Participants were given 2 points for a fully accurate, abstract interpretation of the proverb in which all aspects of the proverb were covered. A 1 point was given for a partially accurate abstract interpretation. A 1 point was also given for an accurate explanation that was somewhat concrete/literal. No points were given for inaccurate interpretations. Fifteen patients and 15 HC’s completed an abbreviated form of the PIT consisting of four proverbs (items 1, 3, 5, and 7, see Appendix A). Thus, to facilitate comparisons, each subject’s raw score on the PIT was converted to a percentage correct score.

The second analysis, the *error analysis*, examined the errors made by the subjects to probe their abstraction ability. Transcripts of the responses of 31 patients and 46 HC’s were retrieved. Errors were divided into three mutually exclusive categories; a partially accurate and abstract response, an accurate or inaccurate concrete response and a non-concrete inaccurate response. Table 3 provides an example from each category of response for one of the proverbs in the PIT. Note here that concrete interpretations could score 1 or 0 depending on the accuracy.

To facilitate categorisation of errors, criteria for concrete responses for each item were created. For example, for the proverb *“the grass is always greener on the other side”* a response was considered concrete if it contained references to another person’s garden or property, the colour of other items or if it was a restatement of the proverb or a single example. These criteria were used to categorise each subject’s responses by the named author (P.M.), who was blind to the presence/absence of a brain lesion for each subject. A random sample (*N*=15) of these categorisations were checked by the named author (K.W.) to assess fidelity with the criteria for concreteness. For each subject the percentage of responses (out of the total number of items on PIT) that fell into each of the three error categories was calculated.

### 2.5 Statistical analysis

The neuropsychological data were screened to ascertain if they were normally distributed and to identify any outliers. We also checked the data for homogeneity of variance. Since error variances differed significantly between the groups for the Trails B Test we transformed these data using a natural log transformation. For the demographic variables in Table 1 we used an ANOVA (age, years of education, months between surgery and assessment, NART IQ) or a chi-square analysis (gender, handedness) to test for a significant group difference. For the data in Tables 4–6b (baseline cognitive tests and accuracy of interpretation of proverbs) an ANCOVA was used for each variable. NART score was entered as a covariate of no interest for the data in Tables 4–6a. Age and NART score were entered as covariates of no interest for the data in Table 6b. When an ANOVA/ANCOVA produced a significant group effect, the between-subjects tests were employed to ascertain the source of the significance. The Dunnett test with a threshold of *p*<0.05 was employed for post hoc analyses. Given the non-parametric nature of the data in Tables 8–10 (error analysis), a Kruskal–Wallis or Mann–Whitney test was used for each variable to test for a significant group difference. Where a significant effect was found a between-subjects Mann–Whitney post hoc test with Bonferroni correction was used to ascertain the source of significance.

Three analyses for grouping frontal patients based on lesion location were carried out.

In the *lateralisation* analysis, *left frontal* and *right frontal* groups were contrasted with controls. This analysis sought to investigate the lateralisation of processes involved in proverb interpretation and figurative language processing. The neuropsychological measures, scores on the PIT and category of error on the PIT were the dependent variables.

A *finer-grained* analysis contrasted *left lateral* versus *right lateral* versus *medial* versus *HC’s.* This analysis investigated the localisation of the processes underpinning proverb interpretation within the frontal cortex. It mirrored those used in previous studies (Reverberi et al., 2005; Robinson et al., 2012; Stuss & Alexander, 2005; Stuss et al., 1998, 2000), allowing comparison to be made with previous behavioural analyses of frontal lobe dysfunction. A comparison of models of cognitive process organisation within the prefrontal cortex (e.g. Duncan, 1995; Stuss & Alexander, 2000) was also possible following this analysis. The orbital group and their respective matched controls were omitted from this analysis due to the small number of patients in the orbital group (*n=6*). Once again, the neuropsychological measures, scores on the PIT and category of error on the PIT were the dependent variables.

A third analysis, a *contrast of specific frontal subgroups*, was based on the procedure of Alexander, Stuss, Picton, Shallice, and Gillingham (2007). This analysis was carried out if and only if a group difference was found on the *finer-grained* analysis. This analysis examined the specificity of any effects found in areas of the frontal cortex. If a frontal group (left or right) or subgroup (left lateral, right lateral or medial) were found to differ significantly from HC’s, then this group was compared with the frontal patients from the subgroup or subgroups that did not differ significantly from HC’s. Scores on the PIT and category of error on the PIT were the dependent variables.

### 2.6 Procedure

Each participant completed the neuropsychological tests, which were administered by experienced clinical neuropsychologists as part of a larger set of tests. All patients underwent an MRI or a CT scan.

## 3 Results

### 3.1 Baseline cognitive tests

#### 3.1.1 Lateralisation analysis

Table 4 details the performance of the three groups on the baseline cognitive measures. There was no main effect of group on performance on the RAPM, with no significant difference between the left and right frontal groups and HC’s. Neither was a significant group effect found on the GNT (see Table 1). For the Stroop Test, group effects were found on the word-reading trial and the colour-naming conflict condition. In both cases left frontal patients alone performed worse than HC’s. A group effect was also found on the Trails B test. Left frontal patients took significantly more time to complete the test than HC’s.

#### 3.1.2 Finer-grained analysis

Table 5 details the performance of the three patient subgroups on the baseline cognitive measures along with the HC’s. As detailed above the orbital subgroup was omitted from each analysis and NART score was included as a covariate.

No significant group effect was found on performance on the RAPM or the Stroop colour-naming conflict condition. Neither was a significant group effect observed for GNT (see Table 2). Group effects were found for the Stroop test word-reading condition. Here, the left lateral subgroup alone performed significantly worse than HC’s. A group effect was also found on the Trails B test, with medial, left lateral and right lateral subgroups all significantly slower than HC’s at completing this test.

### 3.2 Proverbs test (PIT)—accuracy of interpretation

#### 3.2.1 Lateralisation analysis

Comparison of left frontal, right frontal and HC groups on the PIT revealed a significant group effect (see Table 6a). Post hoc comparisons revealed that the left frontal group performed significantly worse than controls on the PIT. The right frontal group narrowly missed out on significance, showing a trend towards a poorer performance on the PIT.

#### 3.2.2 Finer-grained analysis

Comparison of medial, left lateral, right lateral and HC groups on the PIT revealed a significant group effect, with post hoc comparisons revealing that the medial subgroup performed significantly worse than HC’s. Left lateral and right lateral subgroups did not differ significantly from HC’s. An additional comparison of left medial and right medial patients found no difference in terms of their performance on the PIT (*F*(1, 15) =0.177; *p*=0.681).

#### 3.2.3 Contrast of specific frontal subgroups

The *finer-grained analysis* revealed a significant difference between the medial subgroup and HC’s. In contrast, no difference was found between the two lateral subgroups and HC’s. We investigated how specific to the medial subgroup the difficulty interpreting proverbs was by comparing their performance on the PIT with the two lateral groups combined. An ANCOVA was used to test for a group effect with age and NART score entered as a covariate. A group effect was observed, with the means indicating that the medial subgroup performed significantly worse on the PIT than the other lateral patients (orbital patients excluded, see Table 6b).

#### 3.2.4 Orbitofrontal subgroup

The orbitofrontal subgroup was excluded from the analysis due to the low number of patients (*n*=6). Qualitatively, it was noted that the mean performance of this subgroup on the PIT was comparable to that of the non-impaired subgroups (orbital group, mean: 59.38% sd: 24.29%; HC’s, mean: 65.50%, sd: 15.60%), with two of the six orbital patients scoring more than 1.5 standard deviations below the mean of the control group.

#### 3.2.5 Correlations with baseline cognitive tests

Given the results above, it is of interest if cognitive dysfunction as revealed by the baseline cognitive tests may predict performance on the PIT. A correlational analysis between PIT performance and each baseline test was carried out to this end for each group and subgroup. The results of each correlation are shown in Table 7. No significant correlations were observed between the performance on the PIT and the RAPM for any patient group or subgroup. Scores on the GNT and the Stroop colour-naming conflict condition correlated significantly with PIT performance for the patients as a whole and for the medial subgroup. On the Trail Making Test Part B, a significant correlation was observed for the left frontal group only. No correlation was noted between the performance on the Stroop word-reading condition and the performance on the PIT for any patient group or subgroup.

#### 3.2.6 Fluid intelligence

Given our interest in the relationship between the performance on the PIT and fluid intelligence, we compared the performance within patient subgroups on the measure of fluid intelligence (RAPM). Although PIT score was seen to be significantly worse in the medial patients compared to lateral patients (see above), they did not differ from lateral patients significantly in terms of performance on the RAPM (*F*(1, 43) =0.556; *p*=0.460). In fact, none of the three frontal subgroups were found to differ from each other on this measure (*F*(2, 42) =0.282; *p*=0.756).

### 3.3 Proverbs test (PIT)—error analysis

Here we investigated if there were differences in terms of abstraction or concreteness in the type of responses given by our patient sample and HC’s. For this purpose, errors were categorised into three mutually exclusive categories as detailed above (see Table 3). Results of group comparisons on these variables are as follows.

#### 3.3.1 Lateralisation analysis

Comparing left frontal, right frontal and HC groups, a significant group effect was found for both concrete responses as a percentage of total errors and partially correct responses as a percentage of total errors. No difference was found between the left frontal, right frontal and HC groups in terms of non-concrete incorrect responses as a percentage of total errors (see Table 8).

Breaking down these effects using post hoc analyses with Bonferroni corrections, it was found that a higher percentage of the left frontal group’s errors were concrete when compared with the HC group. Right frontal patients and HC’s did not differ from each other in terms of the percentage of errors that were concrete. A significantly lower percentage of the left frontal patients’ errors were partial errors compared to HC’s. No difference was found between right frontal patients and HC’s in terms of the percentage of errors that were partial errors.

#### 3.3.2 Finer-grained analysis

**Fig. 4:**
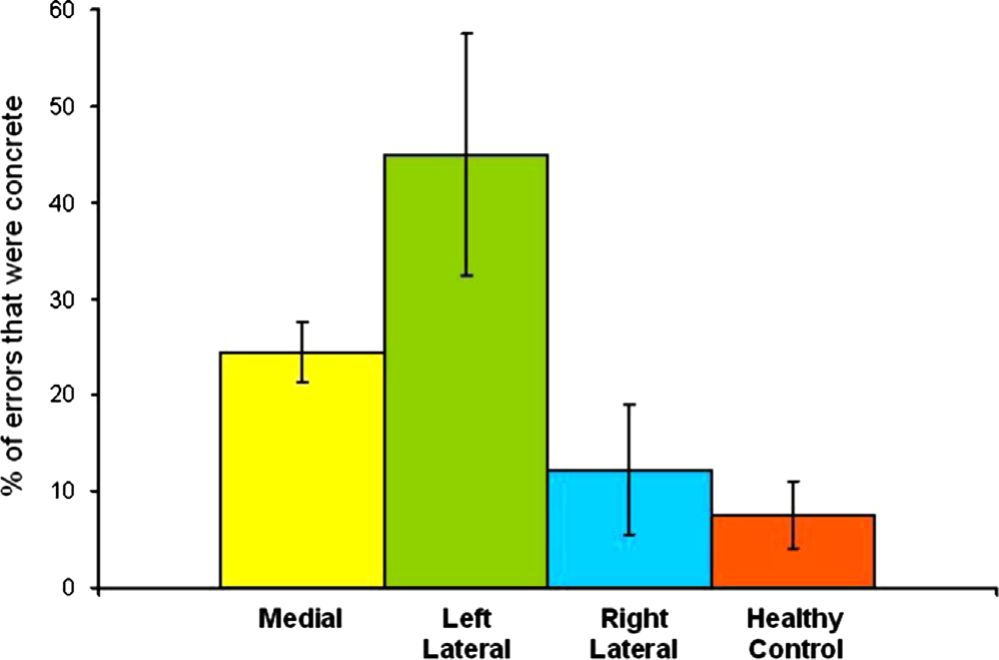
Percentage of total errors made by patient subgroups that were concrete errors.

Repeating this analysis of errors for the frontal subgroups, significant group differences were found for the percentage of errors that were concrete responses and partially correct responses (see Table 9 and Fig. 4).

Using post hoc analyses with Bonferroni correction, it was found that for the left lateral subgroup alone, a significantly higher percentage of their errors were concrete responses when compared with HC’s. Medial and right lateral subgroups did not differ significantly from HC’s in this respect. As depicted in Fig. 4, the left lateral group was almost twice as likely as medial patients and more than three times more likely than right lateral patients to make a concrete response when making an error. This result is further emphasised if one examines the likelihood of 50% or more of errors being concrete responses. The three patient groups differed in this regard (χ^2^(2, *N*=28)=0.049), with a post hoc Fisher’s exact test demonstrating that the left lateral patients were significantly more likely than the other two subgroups to make concrete responses on 50% or more of their errorful responses (*p*=0.029).

For the left lateral and medial subgroups, a significantly lower percentage of their errors were partially correct when compared with HC’s with no significant difference found between the right lateral subgroup and HC’s in this regard.

#### 3.3.3 Contrast of specific frontal subgroups

Here we examined if the patterns of concrete, partial and non-concrete incorrect errors in the Medial subgroup would differ from the other two frontal subgroups. Right and left lateral subgroups were combined for this analysis and compared with the medial subgroup based on the three categories of error (see Table 10). No group difference was observed for percentage of concrete responses, partially correct responses, or non-concrete incorrect responses.

#### 3.3.4 Orbitofrontal subgroup

Qualitatively, it was noted that none of the three of six orbitofrontal subgroup patients for whom error data were available made a concrete error on the PIT. Only one patient made a single non-concrete incorrect error. The percentage of errors made by the orbital group that were partial errors was comparable to controls (orbital group, mean: 86.77%, sd: 22.91%; HC’s, mean: 79.25%, sd: 25.64%).

## 4 Discussion

### 4.1 Summary of results

To our knowledge, this is the first study to investigate the performance of a large number of non-aphasic patients with focal frontal lesions on a proverb interpretation task (PIT). The PIT requires an abstract interpretation of proverbs that relates to a wide variety of contexts, as opposed to a literal, concrete interpretation. Our samples of frontal patients and healthy controls (HC’s) were matched for age and on a measure of estimated pre-morbid intellectual ability. They did not differ from controls in terms of gender or performance on a test of nominal skills. Also, the patient groups and subgroup did not differ in terms of the number of frontal regions damaged, the time between injury and assessment and the likelihood of subcortical damage. However, significant differences were found on tasks sensitive to executive dysfunction, as well as on a fluid intelligence task. The patients’ performance on the PIT was analysed as follows.

#### 4.1.1 Lateralisation analysis

This provides data relevant to previous studies investigating the role of the left and right frontal lobes in proverb interpretation. This analysis showed a significant impairment on the PIT for the left frontal patients and a trend towards impairment in the right frontal group. Previous studies have reported impairment in proverb interpretation following lesions to either the left or right frontal lobes (Benton, 1968; Roca et al., 2010).

#### 4.1.2 Finer-grained analysis

This provides a *potential theoretical interpretation* for the data. It focussed on the role of medial and lateral frontal areas in proverb interpretation. This analysis showed that only patients with medial frontal lobe damage were significantly impaired on the PIT relative to HC’s.

#### 4.1.3 Contrast of specific subgroups

This compared directly the performance of the medial frontal subgroup with the lateral subgroups combined. Quite strikingly, the medial frontal subgroup performed significantly worse on the PIT than the lateral patients combined.

#### 4.1.4 Error analysis

We analysed the patients’ and HC’s responses in terms of the percentage of their errors that were concrete, partially correct and non-concrete incorrect. We found that:

1. For the left lateral patients alone, a significantly higher percentage of their errors were concrete when compared to HC’s.
2. For the medial and left lateral patients, a lower percentage of their errors were partially correct when compared to HC’s.

In addition to these results, none of our patient groups or subgroups performed worse than HC’s on a measure of fluid intelligence (RAPM). Moreover, fluid intelligence did not correlate with the performance on the PIT in any of our patient groups or subgroups.

### 4.2 Proverb interpretation and the medial frontal lobe

Patients with medial frontal lesions were significantly impaired on the PIT compared to HC’s and lateral patients. This finding suggests that medial frontal areas play a significant role in proverb interpretation. This is in line with previous imaging findings. For example, Wallentin et al. (2005) reported higher medial frontal activity during the comprehension of more abstract sentences rather than concrete sentences. Moreover, increased activity in medial frontal areas has been shown to be associated with verbal analogical reasoning, which, as outlined in the introduction, shares many of the features of proverb interpretation (Green et al., 2010, 2012).

One possible explanation for the medial effect is in line with the interpretation of Romero Lauro et al. (2008). In this study the authors suggested that when comprehending idioms, two interpretations of the sentence are available, idiomatic and literal. According to the authors, the anterior medial prefrontal cortex is involved in selecting the less natural interpretation (i.e. the idiomatic interpretation). However, the anterior medial prefrontal region activated in the Romero Lauro et al. study (−4, 54, 32) is very close to that area found by Gilbert, Frith, and Burgess (2005) to be more activated in stimulus-oriented rather than stimulus-independent processes, which makes this interpretation somewhat less plausible; the less natural interpretation is presumably not the result of a more stimulus-oriented process.

An alternative possibility is derived from the hypothesis that the medial frontal cortex plays a key role in attentional “energisation”. This is a top-down process that acts to initiate and sustain one specific type of thought process or behaviour rather than another (Stuss & Alexander, 2007), particularly in situations where the responses are not over-learned (Shallice et al., 2008). Proverb interpretation requires generation of not over-learned responses. To make a correct interpretation a proverb will activate abstract as well as concrete interpretations. However, correct responses on the PIT would require one to select a weaker abstract interpretation over a more dominant concrete one. Given this, the difficulty shown by our medial patients on the PIT may reflect an energisation difficulty.

### 4.3 Proverb interpretation and the left lateral frontal lobe

Our left lateral subgroup did not differ from HC’s in terms of their overall level of performance on the PIT. However, of the errors they made a significantly higher percentage were concrete errors, accounting for 44.92% of their total errors. This is in contrast to the other frontal subgroups, for whom 24.46% (medial) and 12.25% (right lateral) of their errors were concrete. This result is consistent with McDonald et al.’s (2008) findings, where left frontal lesions were associated with poorer abstraction compared to right frontal lesions. We will argue later that concrete errors are at least as sensitive a measure of frontal “executive” impairment as overall performance.

Left lateral frontal areas have previously been linked with figurative language processes in imaging studies with the main emphasis being on the left inferior frontal gyrus (Rapp et al., 2004; Romero Lauro et al., 2008; Stringaris et al., 2007; Yang et al., 2010). It has been suggested that this region is involved in selection between competing verbal responses (see e.g. Robinson, Blair, & Cipolotti, 1998; Thompson-Schill et al., 1997). A related although more general hypothesis is that the left inferior frontal gyrus is involved in “difficult” semantic operations per se (e.g. Yang et al., 2010). Neither of these hypotheses explains why, unlike the medial impairment, the left lateral impairment of our patients does not manifest itself in an overall poorer performance but specifically in a higher rate of concrete errors. This result suggests a third possible explanation related to a hypothesis put forward by Shallice and Cooper (2011, submitted for publication) for abstract words. This hypothesis suggests that the left inferior frontal gyrus is involved in constructing or storing abstract representations. Damage to this region would leave only the literal interpretation available to the patient. However, further investigations are required to disambiguate between these three types of explanation.

We would like to argue that the performance of the left lateral subgroup has implications for the clinical use of the PIT. Our left lateral patients were on average 2 standard deviations worse than HC’s in terms of the percentage of errors that were concrete. The medial subgroup was on an average more than 1 standard deviation worse. By contrast, only the medial subgroups were more than 1 standard deviation worse than HC’s in terms of their overall performance on the PIT. Thus, large differences were found between two subgroups and HC’s in terms of concrete errors, whereas only the medial subgroup differed significantly from HC’s in terms of overall performance on the PIT. Therefore, in clinical scenarios it would appear that concrete responses on the PIT rather than overall performance are likely to represent a more sensitive measure of frontal executive dysfunction.

### 4.4 Lateralisation of figurative language processing

Studies of figurative language processes have provided somewhat inconclusive evidence regarding the lateralisation of anatomical substrates involved in figurative language processes. Thus, some studies suggested that bilateral frontal areas are crucial for idiom (e.g. Rizzo et al., 2007; Zempleni et al., 2007; see Thoma & Daum, 2006 for review) and proverb interpretation (Benton, 1968). Other studies have suggested that left frontal areas are critical for this process (e.g. Cacciari et al., 2006; Hillert & Buračas, 2009; Papagno et al., 2006; Romero Lauro et al., 2008). Several other authors have ascribed a crucial role for right hemisphere regions in figurative language processes or in suppressing literal interpretations of figurative language (e.g. see Bookheimer, 2002 for review; Tompkins & Lehman, 1998). Recently, Roca et al. (2010) found some evidence for right anterior frontal involvement in proverb interpretation. Unfortunately, the types of errors made by their patients were not reported.

Our finding that patients with left and right medial frontal lobe lesions were significantly impaired on the PIT seems to provide some limited support for the suggestion that the frontal lobes bilaterally are implicated in the processing of figurative language. With regards to a right-hemisphere advantage in processing figurative language, we failed to find evidence for increased numbers of concrete responses following right frontal lobe lesions. Indeed, our error analysis differentiated left frontal patients, but not right frontal patients, from HC’s.

Our results are therefore in conflict with the hypothesis that the right hemisphere has a particular role in figurative language processes (e.g. Bookheimer, 2002; Giora et al., 2000; Stringaris et al., 2006; Tompkins & Lehman, 1998). Instead, our data suggest that an impaired performance on the PIT is associated with bilateral medial frontal lobe lesions and that an increased number of concrete responses are associated with the damage to left lateral areas of the frontal lobes. Our data also suggest that separate areas of the frontal lobes may contribute differentially to figurative language processing.

### 4.5 Fluid intelligence and proverb interpretation

The performance of our frontal patients on the PIT did not correlate with the performance on a fluid intelligence test (RAPM). Roca et al. (2010) found this correlation, but only when combining patient and control groups as a whole. However, the authors found that this correlation could not entirely explain performance on the PIT in their frontal sample, which is corroborated by our study. Indeed, Roca et al. also examined the mean of the residual deficits not accounted for by fluid intelligence deficits on five tasks, including the PIT. They reported that right anterior frontal lesions were associated with the mean of the residual deficits. Furthermore; Roca et al. did not find a difference in performance on the PIT when comparing their four frontal subgroups. In contrast, the only group impaired relative to HC’s in our study (medials) was also impaired relative to the other two frontal subgroups (left and right laterals).

There are some notable differences between our study and that of Roca et al. Whereas Roca et al. had 15 frontal lobe patients completing their version of the PIT, of which less than five were medial patients, our study had 52 frontal patients of whom 17 were medial. Our increased numbers of patients would be expected to lead to a more sensitive study. Secondly, the inclusion of patients and healthy controls in the correlations in Roca et al. (2010) is a possible source of divergent findings. There were also some important differences between the scoring procedures adopted to analyse responses on the PIT in Roca et al.’s study and in our study. Roca et al. scored responses as correct, an example and incorrect whereas we have used a more differentiated scoring procedure. It is possible that our scoring criteria were more stringent and therefore may have highlighted difficulties in frontal patients that were not revealed by Roca et al. This is supported by the observation that the mean performance for the HC’s in our study (65.50%) is lower than that of the HC’s tested in Roca et al.’s study (91.33%).

One weakness of our study was the small number of patients with orbitofrontal cortex damage, which led to their exclusion from the finer-grained analysis. Functional imaging studies have linked this area of the frontal lobes with analogical verbal reasoning, a cognitive process sharing many of the features of proverb interpretation (Bunge et al., 2005; Green et al., 2006). Future studies could examine proverb interpretation in larger numbers of orbitofrontal patients. A second weakness was that for our retrospective study additional data from language tasks other than nominal tasks were not available. This has prevented us from investigating whether the impairments in frontal patients on the PIT could be linked with other broader language processes.

A third weakness of our study was the information available on lesion extent for our patient sample. Although we found no difference between the frontal subgroups in terms of the number of frontal regions damaged, we were unable to compare each group in terms of the overall volume of cortex damaged by the lesions. To obtain a large enough sample, 12 years of patient referrals needed to be included and in the earlier patient referrals the clinical scans available were not suitable for VBM or VLSM procedures. It is therefore possible that subgroups differed in terms of total lesion volume, which may have contributed to the differences reported between the groups on the PIT.

Our results show for the first time that medial and left lateral areas play a crucial role in proverb interpretation. We have also demonstrated that medial frontal lesions impair performance on the PIT, with left lateral lesions giving rise to significantly increased levels of concrete errors compared with HC’s. Our results do not support the view that deficits in the PIT following focal frontal lobe lesions reflect a deficit in fluid intelligence. Our findings also fail to support accounts that stress a specific role for the right frontal lobe in figurative language processing. Instead, our results seem to support the notion that proverb interpretation engages a set of specialised cognitive processes underpinned by medial and left lateral areas.

## Figures and Tables

**Table A1:** Patient aetiology (*N*=48).

**Aetiology**	**Left frontal**	**Right frontal**
Sub-arachnoid haemorrhage	3	2
Stroke	1	2
Haematoma	0	1
ACoAneurysm	1	1
Astrocytoma unspecified grade	1	1
Glioma unspecified grade	1	0
Glioma—low grade	0	4
Glioma—grade II	1	0
Meningioma unspecified grade	2	5
Meningioma—grade I	1	0
Meningioma—grade II	1	0
Oligodendroglioma unspecified grade	1	1
Oligodendroglioma—grade II	0	2
Oligodendroglioma—grade III	1	0
Glioblastoma unspecified grade	1	2
Glioblastoma—grade IV	2	0
Oligoastrocytoma	5	0
Metastasis	2	0
Tumour unspecified	3	0

**Table 1:** Demographic characteristics—left and right frontal groups and HC’s.

	**Left frontal*****N*****=29 mean** (**SD**)	**Right frontal*****N*****=23 mean** (**SD**)	**HC*****N*****=52 mean** (**SD**)	**Group comparison*****F*****-value**
Age (years)	46.48 (11.26)	45.61 (15.76)	47.35 (14.59)	0.128 (*p*=0.880)
Gender (male/female)	17/12	11/12	25/27	0.944^a^ (*p*=0.624)
No. months between surg/VA/neuropsy^b^	18.16 (26.05)	22.77 (34.22)	N/A	0.286 (*p*=0.595)
No. of brain regions damaged	3.11 (2.35)	2.70 (1.66)	N/A	0.500 (*p*=0.486)
GNT (no. correct/30)	20.76 (3.99)	22.35 (3.97)	22.77 (3.96)	2.438 (*p*=0.092)
NART IQ	106.10 (15.12)	111.48 (8.95)	110.85 (9.83)	1.990 (*p*=0.142)

SD, standard deviation; HC, healthy controls; no., number; surg, surgery; VA, vascular accident; neuropsy, neuropsychological assessment; N/A, non applicable; GNT, Graded Naming Test; NART, National Adult Reading Test. All significant comparisons in bold.

a Chi-squared analysis.

b Number of cases missing (left frontal=3).

**Table 2:** Demographic characteristics—three prefrontal subgroups and HC's.

	**Medial*****N*****=17 mean** (**SD**)	**Left lateral*****N*****=16 mean** (**SD**)	**Right lateral*****N*****=13 mean** (**SD**)	**HC*****N*****=46 mean** (**SD**)	**Group comparison*****F*****-value**
Age (years)	42.29 (10.04)	47.44 (12.78)	46.46 (14.74)	48.38 (14.41)	0.560 (*p*=0.643)
Gender (male/female)	7/10	10/6	6/7	20/26	2.006^a^ (*p*=0.571)
No. months between surg/VA/neuropsy^b^	32.71 (39.84)	18.27 (27.71)	11.27 (18.94)	N/A	1.849 (*p*=0.171)
No. of brain regions damaged	2.36 (1.66)	4.06 (2.74)	3.23 (2.28)	N/A	2.366 (*p*=0.106)
NART IQ	109.18 (11.21)	104.31 (17.81)	112.00 (8.803)	110.64 (9.22)	1.459 (*p*=0.231)
GNT (no. correct/30)	22.59 (4.39)	19.94 (3.70)	22.15 (3.46)	22.50 (3.84)	2.074 (*p*=0.109)

SD, standard deviation; HC, healthy controls; no., number; surg, surgery; VA, vascular accident; neuropsy, neuropsychological assessment; N/A, non applicable. All significant comparisons in bold.

a Chi-squared analysis.

b Number of cases missing (medial=1/left lateral =1).

**Table 3:** Scoring examples for “Rome wasn′t built in a day” proverb.

**Sample response**	**Score**
“It takes patience and time to complete a great, worthwhile project” (***Fully accurate and abstract***)	2
“Things take time, but you will get there in the end” (***Partially accurate and abstract***) “Any great city or empire, like Rome, will not be completed overnight” (***Accurate concrete***)	1
“Quit while you are ahead” (***Non-concrete inaccurate***) “Rome is a beautiful city” (***Inaccurate concrete***) Don′t know	0

**Table 4:** Performance on baseline cognitive tests—left and right frontal groups and HC's.

	**Left frontal*****N*****=29 mean** (**SD**)	**Right frontal*****N*****=23 mean** (**SD**)	**HC*****N*****=52 mean** (**SD**)	**Group comparison*****F*****-value**
RAPM (number correct/12)^a^	8.00 (2.69)	8.83 (2.08)	9.28 (1.66)	2.116 (*p*=0.126)
Trails B—time in msec^b^	**109.96** (**99.44**)⁎ (***p*****=0.009**)†	90.46 (68.26) (*p*=0.109)	66.45 (21.48)	**3.881** (***p*****=0.026**)
Stroop word—no. words^c^	**206.62** (**70.64**)⁎ (***p*****=0.013**)†	224.07 (50.30) (*p*=0.145)†	244.07 (48.96)	**3.402** (***p*****=0.037**)
Stroop colour—no. colours^d^	**80.12** (**39.78**)⁎ (***p*****=0.003**)†	95.60 (36.43) (*p*=0.168)†	106.74 (23.72)	**4.027** (***p*****=0.010**)

SD, standard deviation; HC, healthy controls; no., number. All significant comparisons in bold.

† Probability value from post hoc test (patient subgroup versus HC’s).

⁎ *p*<0.05 (significant group difference between patient group and HC’s).

a Number of cases missing (HC=2).

b Number of cases missing (left frontal=2/HC=5).

c Number of cases missing (left frontal=2/right frontal=1/HC=1).

d Number of cases missing (left frontal=6/right frontal=2/HC=1).

**Table 5:** Performance on baseline cognitive tests—three prefrontal subgroups and HC's.

	**Medial*****N*****=17 mean** (**SD**)	**Left lateral*****N*****=16 mean** (**SD**)	**Right lateral*****N*****=13 mean** (**SD**)	**HC*****N*****=46 mean** (**SD**)	**Group difference*****F*****-value**
RAPM (number correct/12)^a^	9.00 (2.76)	7.63 (2.60)	8.46 (2.30)	9.36 (1.63)	1.898 (*p*=0.136)
Trails B—time in msec^b^	**85.26** (**56.90**)⁎ (***p*****=0.026**)†	**129.88** (**125.00**)⁎ (***p*****=0.001**)†	**102.59** (**83.75**)⁎ (***p*****=0.012**)†	66.62 (21.44)	**6.005**⁎ (***p*****=0.001**)
Stroop word—no. words^c^	221.52 (53.58) (*p*=0.200)†	**193.27** (**74.14**)⁎ (***p*****=0.007**)†	214.74 (47.56) (*p*=0.108)†	248.59 (57.10)	**2.928**⁎ (***p*****=0.038**)
Stroop colour—no. colours^d^	101.61 (30.49)	80.50 (44.63)	85.22 (41.63)	105.95 (23.46)	2.371 (*p*=0.077)

SD, standard deviation; HC, healthy controls; GNT, Graded Naming Test; no., number; msec, milliseconds; RAPM, Raven’s Advanced Progressive Matrices. All significant comparisons in bold.

† Probability value from post hoc test (patient subgroup versus HC’s).

⁎ *p*<0.05 (significant group difference between patient group and HC’s).

a Number of cases missing (healthy controls=2).

b Number of cases missing (left lateral=2/healthy controls=5).

c Number of cases missing (medial=1/left lateral=1/right lateral=1/healthy controls=1).

d Number of cases missing (medial=2/left lateral=4/right lateral=1/healthy controls=1).

**Table 6a:** Proverb interpretation task accuracy—comparison of left and right frontal groups and frontal subgroups with HC’s.

**Lateralisation analysis**^a^	**Left frontal*****N*****= 27 mean** (**SD**)	**Right frontal*****N*****=23 mean** (**SD**)		**HC*****N*****=52 mean** (**SD**)	**Group difference*****F*****-value**
Score on proverbs task (% score)^b^	**53.02** (**23.18**)⁎ (***p*****=0.003**)†	57.34 (19.46)^c^ (*p*=0.059)†		65.50 (15.60)	**3.400**⁎ (***p*****=0.020**)

**Finer**-**grained analysis**	**Medial*****N*****=17 mean** (**SD**)	**Left lateral*****N*****=16 mean** (**SD**)	**Right lateral*****N*****=13 mean** (**SD**)	**HC*****N*****=46 mean** (**SD**)	**Group difference*****F*****-value**

Score on proverbs task (% score)^b^	**48.53** (**16.76**)⁎ (***p*****=0.001**)†	56.25 (23.27) (*p*=0.195)†	59.62 (24.02) (*p*=0.154)†	66.71 (15.60)	**4.220**⁎ (***p*****=0.008**)

SD, standard deviation; HC, healthy controls. All significant comparisons in bold.

† Probability value from post hoc test (patient subgroup versus HC’s).

⁎ *p*<0.05 (significant group difference between patient group and HC’s).

a Orbital patients and their matched HC’s included.

b Fifteen healthy controls and 15 patients were administered four items on the proverbs task, all others were administered eight items. Score represents the patient’s score on the proverbs task expressed as a percentage. See text for guide to method of scoring.

c Approaching significance (*p*=0.059).

**Table 6b:** Proverb interpretation task accuracy—comparison of medial and lateral frontal subgroups.

	**Medial*****N*****=29 mean** (**SD**)	**Lateral frontal*****N*****=29 mean** (**SD**)	**Group difference*****F*****-value**
Score on proverbs task (% score)^a^	48.53 (16.76)	57.76 (23.25)	**4.296**⁎ (***p*****=0.044**)

SD, standard deviation. All significant comparisons in bold.

⁎ *p*<0.05 (significant group difference between patient group and HC’s).

a Eighteen healthy controls and 22 patients were administered four items on the proverbs task, all others were administered eight items. Score represents the patient’s score on the proverbs task expressed as a percentage. See text for guide to method of scoring.

**Table 7:** Pearson’s *r* coefficients for correlations of performance on the PIT with the performance on baseline cognitive measures.

	**All frontal patients**	**Left frontal**	**Right frontal**	**Left lateral**	**Right lateral**	**Medial**
RAPM	0.170 (*p*=0.229)	0.244 (*p*=0.203)	−0.016 (*p*=0.942)	0.316 (*p*=0.233)	0.092 (*p*=0.764)	0.321 (*p*=0.209)
Trails B—time in msec	−0.271 (*p*=0.057)	−**0.396**⁎ (***p*****=0.041**)	0.022 (*p*=0.921)	−0.475 (*p*=0.086)	−0.027 (*p*=0.931)	−0.318 (*p*=0.213)
Stroop word—no. words	0.106 (*p*=0.469)	0.255 (*p*=0.200)	−0.247 (*p*=0.267)	0.269 (*p*=0.332)	−0.295 (*p*=0.352)	0.146 (*p*=0.590)
Stroop colour—no. colours	**0.311**⁎ (***p*****=0.040**)	0.383 (*p*=0.072)	0.182 (*p*=0.429)	0.415 (*p*=0.180)	0.338 (*p*=0.283)	**0.619**⁎ (***p*****=0.014**)
Graded Naming Test	**0.344**⁎ (***p*****=0.012**)	0.365 (*p*=0.051)	0.285 (*p*=0.187)	0.441 (*p*=0.087)	0.275 (*p*=0.363)	**0.586**⁎ (***p*****=0.013**)

⁎ *p*<0.05 (significant correlation).

**Table 8:** Category of errors^a^ made by left frontal and right frontal patients and HC's.

	**Left frontal*****N*****=16 mean** (**SD**)	**Right frontal*****N*****=15 mean** (**SD**)	**HC*****N*****=37 mean** (**SD**)	***χ***^**2**^ (***p*****-value**)
Concrete errors as % of total errors	**36.11** (**38.10**)* (***p*****=0.009**)†	13.17 (22.24) (*p*=0.224)†	8.31 (19.65)	**9.36**5* (***p*****=0.001**)
Partial errors as % of total errors	**40.48** (**32.78**)* (***p*****=0.001**)†	59.60 (36.21) (*p*=0.066)†	79.25 (25.64)	14.371* (***p*****=0.006**)
Non-concrete incorrect errors as % of total errors	17.16 (19.19)	20.56 (26.82)	12.44 (18.90)	1.376 (*p*=0.503)

All significant comparisons in bold.

† Probability value from post hoc test (patient subgroup versus HC’s).

⁎ *p*<0.05 (Bonferroni corrected for comparisons between individual patient groups and HC’s).

a See Materials and methods section for complete explanation of response categories.

**Table 9:** Category of errors^a^ made by left lateral, right lateral and medial frontal patients and healthy controls.

	**Medial*****N*****=8 mean** (**SD**)	**Left lateral*****N*****=11 mean** (**SD**)	**Right lateral*****N*****=9 mean** (**SD**)	**HC*****N*****=33 mean** (**SD**)	***χ***^**2**^ (***p*****-value**)
Concrete errors as % of total errors	24.46 (8.71) (*p*=0.062)†	**44.92** (**41.73**)⁎ (***p*****=0.001**)†	12.25 (20.32) (*p*=0.526)	7.59 (20.11)	**11.841**⁎ (***p*****=0.008**)
Partial errors as % of total errors	**42.89** (**29.27**)⁎ (***p*****=0.002**)†	**34.90** (**34.23**)⁎ (***p*****=0.007**)†	61.60 (36.00) (*p*=0.355)	80.22 (24.81)	**16.232**⁎ (***p*****=0.001**)
Non-concrete incorrect errors as % of total errors	35.74 (29.27) (*p*=0.024)	11.09 (14.63) (*p*=0.979)	15.04 (20.39) (*p*=0.763)	12.19 (19.03)	6.408 (*p*=0.093)

All significant comparisons in bold.

† Probability value from post hoc test (patient subgroup versus HC’s).

⁎ *p*<0.05 (Bonferroni corrected for comparisons between individual patient groups and HC’s).

a See Materials and methods section for complete explanation of response categories.

**Table 10:** Category of errors^a^ made by medial and lateral frontal subgroups.

	**Medial*****N*****=8 mean** (**SD**)	**Lateral frontal*****N*****=20 mean** (**SD**)	*Z* (*p*-value)
Concrete errors as % of total errors	21.38 (24.64)	30.22 (36.99)	−0.215 (p=0.830)
Partial errors as % of total errors	42.89 (29.27)	46.91 (36.72)	−0.051 (p=0.980)
Non-concrete incorrect errors as % of total errors	35.74 (29.27)	12.87 (17.08)	−2.065 (p=0.049)

a See Materials and methods section for complete explanation of response categories.

## Appendix A

See Table A1.

## Appendix B

Proverbs test.

1. Don′t cry over spilt milk.
2. Rome wasn′t built in a day.
3. Where there′s a will there’s a way.
4. Strike while the iron is hot.
5. The grass is always greener on the other side.
6. Let sleeping dogs lie.
7. All that glitters is not gold.
8. Too many cooks spoil the broth.
